# Editorial: Is the singularity near? Causal inference in sport consumer behavior research

**DOI:** 10.3389/fpsyg.2023.1139910

**Published:** 2023-06-09

**Authors:** Kevin K. Byon, James Du, Do Young Pyun

**Affiliations:** ^1^Department of Kinesiology, Indiana University, Bloomington, IN, United States; ^2^Department of Sport Management, Florida State University, Tallahassee, FL, United States; ^3^Department of Sport Marketing and Management, Loughborough University, Loughborough, United Kingdom

**Keywords:** sport consumer behavior research, causal inference, scientific rigor, Bayesian, experimental design

Since its emergence in 1980s, sport consumer behavior research (SCBR) has witnessed exponential growth in popularity and complexity over the past two decades (Funk, 2017). Yet, the current state of knowledge has given little explicit reflection on more inclusive and diverse research settings, cultures, and populations to collect data and develop theory (Delia et al., 2022). With the proliferation of digital information about and globalization of sport organizations, fans, players, virtual connections, and interactions, the field is reaching an inflection point at which access to extensive quantities of data, novel contexts and technologies, and advanced statistical methods are readily available. These new environments enable sport consumer behavior scholars to retest and falsify the conventional assumptions and established frameworks, and to understand previously intractable problems rooted in differences in culture and market economies (Byon and Zhang, 2019; Cunningham et al., 2021).

From a philosophy of science perspective, two complementary approaches dominate the practice of contemporary knowledge construction and diffusion in SCBR: hypothetico-deductive reasoning and observational-inductive reasoning (Mahootian and Eastman, 2009). Assuming the singularity of truth to be explored, researchers who follow the post-positivist paradigms subscribe to the deductive “scientific” approach of applying and falsifying existing theories through hypothesis development and testing (Denzin and Giardina, 2008). The alternative empirics-inductive model, on the contrary, calls for scholars to examine context-specific factors that enable the development of new insights into inquiries of phenomena (Golder et al., 2022). To this end, this Research Topic echoes James (2018) Zeigler's lecture to call for a need to embrace both the breadth and depth of philosophical paradigms in guiding the conduct of scientific inquiries through employing different epistemological and ontological principles. Without a systematic endeavor to expand the boundary applicable to different sociocultural economic settings, the field will be challenged in further cultivating and advancing pertinent theoretical development (Delia et al., 2022). Consistent with the above aims, this Research Topic sought contributions that shed light on broad perspectives of SCBR and offer a bridge to demonstrate how new research contexts combined with methodological robustness can add heuristic value in advancing knowledge discovery in SCBR (Funk, 2019). Selected are five articles that address various sport consumer behavior topics that would help further our understanding of sport consumer behavior.

The motivation behind the first article, entitled *Self-Serving Bias in Performance Goal Achievement Appraisals: Evidence from Long-Distance Runners* is derived from the authorship team's own experience of observing the considerable differences between participants' official finish time and their post self-reported finish time at a long-distance running event. Grounded in self-serving bias, Hyun et al. authors examined how long-distance runners appraise their athletic performance after the event and how those with unsatisfactory race results cope with performance failure. The authors found that record-high-missed runners tend to report more positively biased finish times than record-high-achieved runners, confirming that runners whose actual performance is worse than their expectation would exhibit a self-serving bias to remove any discomfort. The study also reports that runners reporting self-serving bias show lower event satisfaction than their counterparts. The findings provide event organizers with useful information on the psychological process of how participants cope with their worse-than-expected performance.

With consideration of the concern that the COVID-19 lockdown would increase sedentary lifestyle, which, in turn, results in poor physical and mental health conditions, the second article in this Research Topic entitled *Changes in Physical Activity and Depressive Symptoms During* Bayesian *COVID-19 Lockdown: United States Adult Age Groups* by Kim et al. examined potential changes in three health-related factors (i.e., physical activity, non-physical-activity health behavior, and depressive symptoms), and how physical activity is related to depressive symptoms before and after the lockdown among various aged people. Two main findings are (a) the participants maintain their physical activity levels after the lockdown despite significant increases in sedentary behaviors, particularly among young and old people groups; (b) decreases in moderate physical activity are associated with a higher level of depressive symptoms. The use of analyses provides significant benefits over conventional inferential statistics, such as the application of informative priors into empirical probabilistic models and robustness to asymptotic assumption, outliers, and sample size.

The third article, entitled *Effects of Game Outcomes and Status Instability on Spectators' Status Consumption: The Moderating Role of Implicit Team Identification* by Chang and Wann, offers a novel insight into when, how, and why spectators engage in status-seeking behavior. Building on the biosocial theory of status, the authors examined the interactions of game outcome and status instability effects on spectators' status-seeking behavior moderated by implicit team identification. A series of experiments confirmed the causal relationship between decisive game outcomes (victory vs. loss) and status consumption. However, counterintuitive findings were also found in the event of close game outcomes. Also, the authors find the interaction effect of implicit team identification on the relationship between game outcomes and status consumption. The study's findings contributed to the sport consumer behavior literature by uncovering the effects of biological motives on status consumption and the conditions under which spectators' status-seeking behavior changes.

The fourth article, entitled *Effect of 2002 FIFA World Cup: Point of Attachment that Promotes Mass Football Participation* by Kang et al. investigated how various points of attachment related to the 2002 FIFA World Cup influenced football participation frequency immediately after the event and the present frequency of football participation in host countries. An online survey collected data from people who consumed the 2002 FIFA World Cup in both host countries (i.e., Korea and Japan). Hierarchical multiple regression revealed that the high level of attachment to the player and coach showed both short-term and long-term football participation. However, the attachment to the national team and football only enhanced short-term participatory consumption. This study contributes to see results can add to the knowledge concerning mega-events' effects by providing empirical evidence supporting the trickle-down effect of a past FIFA World Cup on mass football participatory consumption in host countries.

Final article entitled *Can Signal Delay and Advertising Lead to Profit? A Study on Sporting* by Wu et al. examined the associations among advertisement/signal delay (stimulus), arousal/attention (organism), and intention to become paying members (response) for live sporting event streaming users in China. Structural equation modeling support that advertisement and delay influenced behavioral intention through arousal and attention. In addition, signal delay exhibited a more substantial indirect effect on behavioral intention over the advertisement. The findings provide critical practical implications with regard to advertising planning and design, better service delivery, and mechanism of arousal and attention.

In conclusion, we hope that the five articles featured in this Research Topic will shed light on the subject of causation in sport consumer behavior research. Although causal inference is not necessarily a requirement of sport consumer behavior research, future studies should acknowledge its merits and be inspired to go beyond mere correlational relationships while exploring a wide spectrum of cultural, demographic, geographical, and socioeconomic segments.

## Author contributions

All authors listed have made a substantial, direct, and intellectual contribution to the work and approved it for publication.
